# Vascular Redox Signaling, Endothelial Nitric Oxide Synthase Uncoupling, and Endothelial Dysfunction in the Setting of Transportation Noise Exposure or Chronic Treatment with Organic Nitrates

**DOI:** 10.1089/ars.2023.0006

**Published:** 2023-05-03

**Authors:** Thomas Münzel, Andreas Daiber

**Affiliations:** ^1^ Department of Cardiology 1, University Medical Center of the Johannes Gutenberg-Universität Mainz, Mainz, Germany.; ^2^ German Center for Cardiovascular Research (DZHK), Partner Site Rhine-Main, Mainz, Germany.

**Keywords:** oxidative stress, superoxide, peroxynitrite, nitric oxide synthase uncoupling, NADPH oxidase, nitrate tolerance, transportation noise exposure

## Abstract

**Significance:**

Cardiovascular disease and drug-induced health side effects are frequently associated with—or even caused by—an imbalance between the concentrations of reactive oxygen and nitrogen species (RONS) and antioxidants, respectively, determining the metabolism of these harmful oxidants.

**Recent Advances:**

According to the “kindling radical” hypothesis, the initial formation of RONS may further trigger the additional activation of RONS formation under certain pathological conditions. The present review specifically focuses on a dysfunctional, uncoupled endothelial nitric oxide synthase (eNOS) caused by RONS in the setting of transportation noise exposure or chronic treatment with organic nitrates, especially nitroglycerin (GTN). We further describe the various “redox switches” that are proposed to be involved in the uncoupling process of eNOS.

**Critical Issues:**

In particular, the oxidative depletion of tetrahydrobiopterin and S-glutathionylation of the eNOS reductase domain are highlighted as major pathways for eNOS uncoupling upon noise exposure or GTN treatment. In addition, oxidative disruption of the eNOS dimer, inhibitory phosphorylation of eNOS at the threonine or tyrosine residues, redox-triggered accumulation of asymmetric dimethylarginine, and l-arginine deficiency are discussed as alternative mechanisms of eNOS uncoupling.

**Future Directions:**

The clinical consequences of eNOS dysfunction due to uncoupling on cardiovascular disease are summarized also, providing a template for future clinical studies on endothelial dysfunction caused by pharmacological or environmental risk factors.

## Introduction

Environmental noise is a major environmental health risk factor (Munzel et al, 2021; Munzel et al, 2018a; Webpage, 2018). A large proportion of the population is exposed to noise levels exceeding the threshold values recommended by the World Health Organization (WHO) guidelines of 55 dB(A), and there is growing evidence linking traffic noise to cardiovascular morbidity and mortality rate (Munzel et al, 2020) and also cerebral disease (Hahad et al, 2019). According to the data of the WHO environmental noise guidelines for the European region, traffic noise is associated with an increased risk of metabolic disease and with a pooled relative risk for ischemic heart disease of 1.08 (95% CI 1.01–1.15) per 10 dB(A) increase in noise exposure, starting at 53 dB(A) (Kempen et al, 2018). Railway and road noise was also associated with increased arterial stiffness, a subclinical marker of atherosclerosis and development of future cardiovascular disease (Foraster et al, 2017).

There is also epidemiological evidence that chronic exposure to road, railway, or aircraft noise is associated with elevated blood pressure, arterial hypertension (in particular nighttime noise) (Munzel et al, 2020), stroke, heart failure, arrhythmia (Munzel et al, 2018a; Munzel et al, 2014a), as well as cardiometabolic disorders such as diabetes and obesity (Munzel et al, 2021). The annual cardiovascular disease burden of noise in Europe is reflected by 1.7 million cases of hypertension, 80,000 hospital admissions, and 18,000 excess deaths (Webpage, 2014). It was estimated that reducing noise levels by 5 dB(A) could reduce hypertension by 1.4% and ischemic heart disease by 1.8%, saving 3.9 billion dollars in health costs (Swinburn et al, 2015).

Babisch et al (2001) have postulated the noise reaction model, where an “indirect pathway,” plays a crucial role in causing cardiovascular diseases (Babisch, 2003). This concept comprises the cognitive perception of noise, the subsequent cortical activation, leading to increased levels of stress hormones that become manifest in neuropsychiatric, metabolic, and cardiovascular diseases (Babisch, 2014; Munzel et al, 2021; Munzel et al, 2018a). Perturbation of the autonomic nervous system and sympathoadrenal activation (Recio et al, 2016), release of proinflammatory mediators, modified (phospho)lipids, activation of leukocytes, oxidative stress, and prothrombotic pathways, and endothelial dysfunction are crucial steps (Munzel et al, 2021; Munzel et al, 2018a; Munzel et al, 2014a).

Recent animal studies support an essential role of oxidative stress, activation and recruitment of immune cells, impairment of the circadian clock and dysregulated gene networks leading to endothelial dysfunction, and vascular/cerebral damage from aircraft noise (Kroller-Schon et al, 2018; Munzel et al, 2017a; Eckrich et al, 2021).

Organic nitrates belong to the class of nitrovasodilators and represent a mainstay in the treatment of stable and unstable angina pectoris and acute and chronic heart failure (Abrams, 1995). The phenomenon of organic nitrate tolerance is a well-recognized clinical problem (Packer, 1989; Katz, 1990; Gori and Parker, 2002). The hemodynamic and anti-ischemic effects of nitroglycerin (GTN), and also other organic nitrates such as isosorbide-5-mononitrate, are lost upon chronic administration due to the rapid development of nitrate tolerance (Munzel et al, 2013; Munzel et al, 2011). Tolerance to nitrates in response to chronic therapy was documented already in the 1970s *in vivo* (Packer et al, 1987; Zelis and Mason, 1975).

Later, the term cross-tolerance was introduced, after establishing that nitrate therapy also impaired endothelial function in experimental animals (Oelze et al, 2013; Munzel et al, 1995b), and also in patients with established coronary artery disease (Gori et al, 2001b; Liuni et al, 2011; Schulz et al, 2002).

We discovered that the pathophysiology leading to nitrate tolerance and cross-tolerance is mainly due to the increased formation of reactive oxygen species (ROS) and reactive nitrogen species in the mitochondria and by different additional ROS-mediated processes, including endothelial nitric oxide synthase (eNOS) uncoupling, nitration/inhibition of prostacyclin synthase, and vascular NADPH oxidase activation (Munzel et al, 2013; Munzel et al, 2011). Oxidative inhibition of the nitrate-bioactivating enzyme mitochondrial aldehyde dehydrogenase (ALDH-2) appears to play a central role for nitrate tolerance in response to GTN (Chen et al, 2002; Sydow et al, 2004).

### Oxidative stress and redox signaling: implications for the cardiovascular system

Increased production of reactive oxygen and nitrogen species (RONS) leading to increased oxidative stress has been demonstrated to be a characteristic concerning many cardiovascular and cerebrovascular diseases (Sies, 2015; Sies, 1991), and also of side effects of drug therapy as exemplified by organic nitrate-induced tolerance (Munzel et al, 2014b; Munzel et al, 2005a) or environmental health risk factors such as transportation noise (Munzel et al, 2017b; Munzel et al, 2017c; Munzel et al, 2014a). Oxidative stress characterizes a state of elevated RONS formation and/or insufficient scavenging of RONS, for example, by low levels of important antioxidant proteins, with a subsequent reduction of NO bioavailability as well as depletion of low-molecular-weight antioxidants, thus causing a shift in the cellular redox balance. The most common RONS include superoxide radicals (^•^O_2_^−^), hydrogen peroxide, hydroxyl radicals, carbon-centered peroxides, peroxyl radicals, nitric oxide (^•^NO) and nitrogen dioxide radicals, peroxynitrite (ONOO^−^), and hypochlorite.

Some of these species act as cellular messengers by conferring redox signaling (Rhee, 1999; Sies, 2017; Stamler, 1994; Ullrich and Kissner, 2006). Nitric oxide (^•^NO) represents a second messenger and is probably the best-characterized radical species that plays a central role in the beneficial regulation of vascular tone and suppression of platelet activation. Xanthine oxidase (XO), NADPH oxidases, uncoupled nitric oxide synthases, and the mitochondrial respiratory chain are the most important biological sources of the superoxide anion (^•^O_2_^−^), an important precursor of many ROS. The detrimental biological role of ^•^O_2_^−^ was further substantiated by the discovery of superoxide dismutases (mitochondrial, Mn-SOD, and cytosolic/extracellular Cu,Zn-SOD), highly specialized enzymes with the sole biological function of detoxifying ^•^O_2_^−^ (McCord et al, 1971).

The extremely fast reaction between ^•^NO and ^•^O_2_^−^ leads to the formation of the highly reactive peroxynitrite (ONOO^−^) (Beckman and Koppenol, 1996). The reaction product from ^•^NO and ^•^O_2_^−^, peroxynitrite, is a significantly stronger oxidant than each of the reaction partners alone and its significant contribution to cardiovascular and cerebrovascular diseases is well established (Ischiropoulos and Beckman, 2003; Turko and Murad, 2002). Since the two molecules react with each other through a diffusion-controlled reaction, in many aspects, ^•^O_2_^−^ can be regarded as a direct antagonist of ^•^NO. In the absence of ^•^NO, ^•^O_2_^−^ is either removed through a spontaneous disproportionation process or it is scavenged by SODs.

In general, in the presence of ^•^NO, the disproportionation of ^•^O_2_^−^ is outcompeted by the formation of ONOO^−^ since the reaction speed between ^•^NO and ^•^O_2_^−^ is threefold to fivefold faster than the disproportionation process of ^•^O_2_^−^ catalyzed by SODs (Beckman and Koppenol, 1996; Kissner et al, 1997). ONOO^−^ leads to comparable redox changes of other biomolecules such as hydrogen peroxide but is regarded ∼1000-fold more potent in causing these redox reactions.

The reactivity of the peroxynitrite anion is quite unique and specific (in the first line oxidation of thiols, thioethers, or metal-catalyzed nitration of tyrosine). Upon protonation, peroxynitrous acid (ONOOH) either isomerizes spontaneously to nitrate (the so-called inactivation route) or it undergoes homolysis to form ^•^NO_2_ and ^•^OH radicals (the so-called toxifying route) that cause one-electron oxidation reactions leading to new products *via* hydroxylation and nitration. The biological existence of ONOO^−^ was demonstrated by the nitration of protein tyrosine residues in atherosclerotic lesions (Beckmann et al, 1994) and other disease conditions (Crow and Beckman, 1995; Ischiropoulos and Beckman, 2003), with prostacyclin synthase nitration and simultaneous inactivation representing a quite specific target under various pathophysiological conditions (Hink et al, 2003; Zou and Bachschmid, 1999; Zou et al, 1999).

**FIG. 1.**
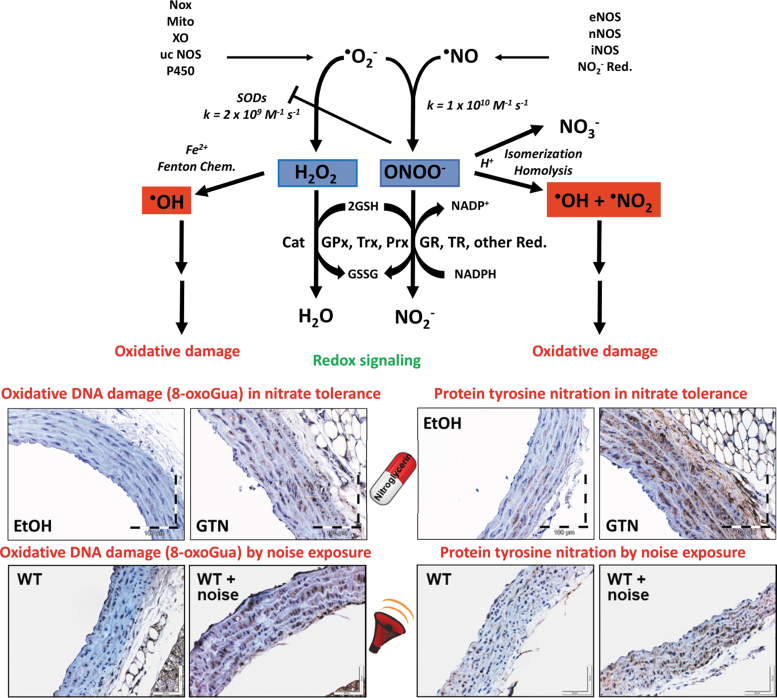
**The chemical basis of vascular oxidative stress and redox signaling by superoxide and nitric oxide in animal models of nitrate tolerance and traffic noise exposure.** *Upper part:* Superoxide (^•^O_2_^−^) formation from Nox, the mitochondrial respiratory chain (Mito), XO, a ucNOS, and P450 side reactions confer redox signaling mainly upon breakdown by self-dismutation or catalyzed by SODs to hydrogen peroxide. Reaction with thiol groups is a major route of detoxification for H_2_O_2_ *via* Prx and Trx or the selenol in GPx—these systems require energy-consuming recycling by NADPH-coupled reductases (GR, TR). Another route for H_2_O_2_ decomposition is catalyzed by catalase (Cat). When H_2_O_2_ accumulates, it may lead to the Fenton reaction (hydroxyl radical formation) upon reaction with ferrous iron (Fe^2+^), triggering severe oxidative damage at the protein, lipid, and DNA levels. Likewise, nitric oxide (^•^NO, previously termed EDRF) is formed from eNOS, nNOS, iNOS, or reduction of nitrite from nutrition conferring the diffusion-controlled reaction with superoxide to yield peroxynitrite anion (ONOO^−^). This rapid kinetics even outcompetes the extremely fast SOD-catalyzed breakdown of superoxide. Upon protonation, peroxynitrite undergoes either spontaneous isomerization to nitrate (representing a detoxifying route) or undergoes homolysis to form the hydroxyl radical (^•^OH) and the nitrogen dioxide (^•^NO_2_) radical leading to oxidative damage comparable with the Fenton reactivity of H_2_O_2_. *Lower part:* (*Left*) Hydroxyl radicals typically induce oxidative DNA damage in the form of 8-oxoGua as envisaged in GTN-treated, nitrate-tolerant rats (GTN with a dose of 50 mg/kg/day for 3.5 days) or aircraft noise-exposed mice [72 dB(A) around-the-clock for 4 days] by immunohistochemistry (*brown* staining). (*Right*) ONOOH and derived free radicals typically induce protein tyrosine nitration as envisaged in GTN-treated, nitrate-tolerant rats or noise-exposed mice by immunohistochemistry (*brown* staining), although MPO/H_2_O_2_/nitrite reaction will also lead to nitration. Scheme in the *upper part* summarized from Daiber and Ullrich (2002) and adapted from Daiber et al (2020c) and Daiber et al (2014) with permission. Images in the *lower part* were reproduced from Mikhed et al (2016) (for nitrate tolerance) and Kvandova et al (2020) (for noise exposure) with permission. 8-oxoGua, 8-oxoguanine; EDRF, endothelium-derived relaxing factor; eNOS, endothelial nitric oxide synthase; GPx, glutathione peroxidase; GR, glutathione reductase; GTN, nitroglycerin; iNOS, inducible nitric oxide synthase; MPO, myeloperoxidase; nNOS, neuronal nitric oxide synthase; Nox, NADPH oxidases; Prx, peroxiredoxins; SODs, superoxide dismutases; TR, thioredoxin reductase; Trx, thioredoxin; ucNOS, uncoupled nitric oxide synthase; XO, xanthine oxidase.

It should, however, be kept in mind that 3-nitrotyrosine is not only an exclusive marker of peroxynitrite reactivity but can be also formed under inflammatory conditions by the reaction of myeloperoxidase (MPO) with hydrogen peroxide in the presence of nitrite leading to the formation of nitrogen dioxide radicals that can nitrate tyrosine (Brennan et al, 2002; Kettle et al, 1997; Sampson et al, 1998). The pathomechanistic features of oxidative stress and adverse redox signaling on the cardiovascular system were previously reviewed in detail (Beckman and Koppenol, 1996; Daiber and Ullrich, 2002; Radi, 2004) and have a significant impact on vascular biology, endothelial function, and importantly, cardiovascular prognosis (Fig. 1) (Daiber et al, 2017c; Heitzer et al, 2001b). This is also reflected by multiple reports on markers of oxidative stress and redox biomarkers in cardiovascular disease (Daiber et al, 2021).

In the present overview, we explain the detrimental impact of oxidative stress on endothelial function in detail for the side effect of chronic treatment with organic nitrates (*e.g.,* GTN), nitrate tolerance (Daiber and Munzel, 2015; Munzel et al, 2013; Munzel et al, 2011), and for exposure to transportation noise (Munzel et al, 2021; Munzel et al, 2018a; Munzel et al, 2018b), an environmental stressor and novel cardiovascular and cerebral health risk factor.

### Oxidative stress, ROS sources, and cardiovascular disease

In the early 1990s, the Harrison's group was the first to describe in an experimental model of hypercholesterolemia that endothelial dysfunction is caused by increased superoxide production due to an activation of the XO (Harrison and Ohara, 1995; Ohara et al, 1993). The molecular proof for a decisive role of oxidative stress in cardiovascular and cerebrovascular diseases was provided by a large number of clinical studies by demonstrating that the acute administration of high dose of the antioxidant vitamin C is able to improve endothelial dysfunction (Daiber et al, 2017c; Heitzer et al, 2001b; Heitzer et al, 1996).

Also, preclinical studies using genetically engineered mice (*e.g.,* knockout mice) elegantly demonstrated the crucial role of ROS producing or degrading enzymes with respect to the initiation and also progression of cardiovascular and cerebral diseases (Daiber and Chlopicki, 2020; Daiber et al, 2020c; Daiber et al, 2017a). For example, the deletion of the NADPH oxidase subunits *p47^phox^* and *Nox-1* has been shown to have a blood pressure lowering effect and to prevent endothelial dysfunction in the model of angiotensin-II-induced hypertension in mice (Landmesser et al, 2002; Matsuno et al, 2005). In addition, GTN- or noise-induced vascular oxidative stress and endothelial dysfunction were markedly improved by genetic deletion of *p47^phox^* and/or *gp91^phox^* (Kroller-Schon et al, 2018; Wenzel et al, 2008).

This is in good agreement with multiple reports on a central role of oxidative stress in causing cardiovascular health side effects in response to chronic GTN treatment (Daiber and Munzel, 2015; Munzel et al, 2013; Munzel et al, 2011) and transportation noise exposure (Munzel et al, 2021; Munzel et al, 2018a; Munzel et al, 2018b). For both the pathophysiological conditions, oxidative DNA damage in the form of 8-oxoguanine lesions, as a footprint of *in vivo* hydroxyl radical formation, and protein tyrosine nitration, as a readout for peroxynitrite *in vivo* formation or MPO/H_2_O_2_/nitrite reactivity, were reported in preclinical studies (Fig. 1) (Mikhed et al, 2016; Kvandova et al, 2020). Likewise, these and other oxidative stress markers were also observed in human samples in response to noise exposure (Hemmingsen et al, 2015; Kroller-Schon et al, 2018) and chronic therapy with organic nitrates (Schulz et al, 2002; McGrath et al, 2002).

According to the concept of “kindling radicals” (or also “bonfire” hypothesis), the initial formation of ROS, for example, *via* the NADPH oxidases, will trigger further damage by causing eNOS uncoupling *via* quite different mechanisms (see “redox switches” below). The ROS-induced ROS production concept, originally postulated for redox activation of mitochondrial reactive oxygen species (mtROS) production by ROS from neighbored dysfunctional mitochondria (Zorov et al, 2000), can now be extended to almost any kind of source of RONS as almost all of these sources contain “redox switches.” Beyond cytoplasmic enzymes, a cross talk between mtROS formation and NADPH oxidases has been established (Daiber, 2010; Schulz et al, 2014; Kimura et al, 2005). For example, mtROS can open the mitochondrial permeability transition pore by redox regulatory mechanisms (Radi et al, 2002).

Upon release of the mtROS into the cytosol (most probably representing H_2_O_2_), these reactive species can activate the redox-sensitive zinc-finger-like complex in the protein kinase C (PKC) (Lin and Takemoto, 2005) and thereby induce the translocation of cytosolic NADPH oxidase subunits of the NOX-1 and NOX-2 isoforms, for example, p47^phox^ or Noxa1, to the membrane thus triggering NADPH oxidase-dependent superoxide release. Prominent examples for this cross talk between mitochondrial and NADPH oxidase-derived ROS were reported for GTN-induced nitrate tolerance (Wenzel et al, 2008) and angiotensin-II-induced hypertension (Dikalova et al, 2010a; Doughan et al, 2008; Kroller-Schon et al, 2014).

Other sources of oxidative stress exhibit comparable redox switches (Daiber et al, 2017a; Schulz et al, 2014): for instance, the conversion of xanthine dehydrogenase to the oxidase form (XO) needs oxidation of critical thiol residues, and uncoupling of eNOS (and also other isoforms) can be mediated by numerous redox switches as discussed below. Of note, not only can the eNOS located in endothelial cells become dysfunctional or uncoupled, but also the eNOS in red blood cells has a significant effect on vascular tone and health (Kuhn et al, 2017; Leo et al, 2021). Of note, eNOS uncoupling and impaired endothelial function were also observed in the setting of nitrate tolerance under GTN therapy (Schulz et al, 2002; Munzel et al, 1995b) and in response to traffic noise exposure (Munzel et al, 2017a; Schmidt et al, 2013).

Of note, increased inducible nitric oxide synthase (iNOS) was reported in the brain of noise-exposed mice, which was prevented by genetic deletion of *Nox-2* (Kroller-Schon et al, 2018). A role of iNOS and arginase enzymes in noise-induced hearing loss was also reported by multiple studies, but the auditory effects of noise are not in the focus of the present review. Nitrate tolerance by GTN treatment was prevented by genetic deficiency or pharmacological inhibition of arginase II (Khong et al, 2012), which may also point to a potential role for iNOS in causing this phenomenon (Wang et al, 2006).

## Redox Switches in eNOS

**FIG. 2.**
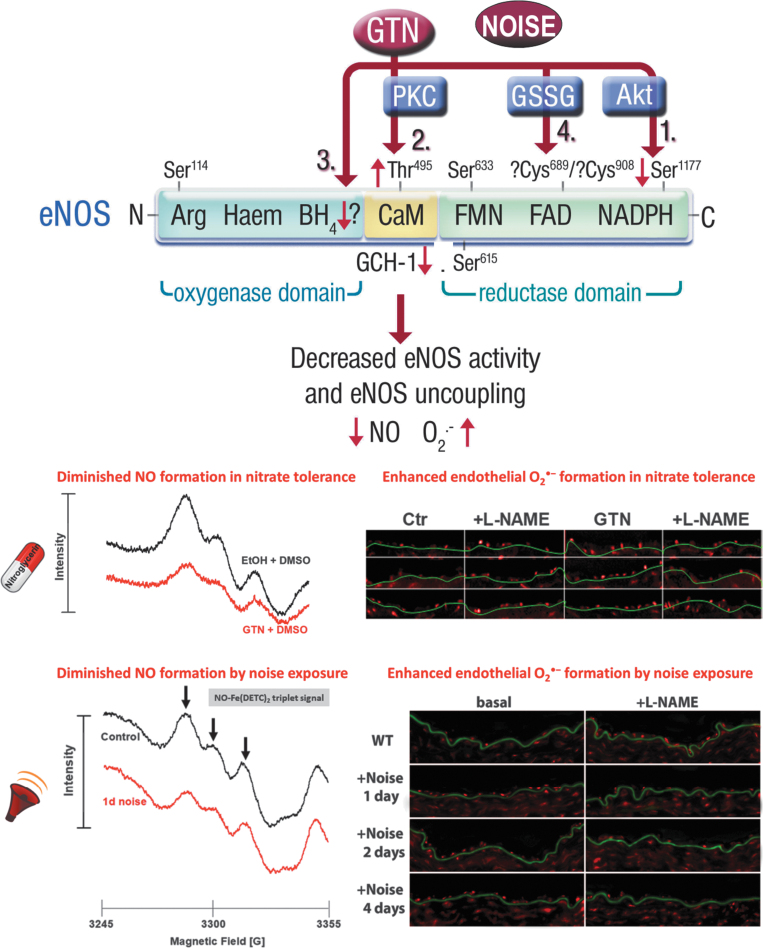
**Proposed mechanisms underlying GTN-induced and traffic noise-mediated eNOS uncoupling and diminished ^•^NO bioavailability.** *Upper part:* GTN treatment causes a decrease in Ser1177 (1) and an increase in Thr495 (2) phosphorylation of the eNOS (Knorr et al, 2011), leading to a decreased activity and uncoupling, respectively. In addition, the key enzyme of the *de novo* synthetic pathway of the eNOS cofactor BH_4_ GCH-1 is downregulated by chronic GTN treatment (3), also leading to a dysfunctional, superoxide (^•^O_2_^−^)-producing nitric oxide synthase. S-Glutathionylation represents another regulatory modification of eNOS, which is increased in the setting of tolerance (Knorr et al, 2011) and upon noise exposure (Munzel et al, 2017a; Steven et al, 2020) (4). *Lower part:* All of these adverse regulatory pathways that are activated by GTN treatment (50 or 100 mg/kg/day for 3.5 days) (Jabs et al, 2015; Knorr et al, 2011) or aircraft noise exposure [72 dB(A) around-the-clock for 1, 2, or 4 days] (Munzel et al, 2017a), including direct scavenging of ^•^NO by ^•^O_2_^−^, lead to diminished ^•^NO bioavailability and enhanced endothelial ^•^O_2_^−^ formation that was blocked by the eNOS inhibitor L-NAME, supporting eNOS uncoupling. “?” nearby Cys^689/908^ means that discrimination between the S-glutathionylation sites is not possible since a pan-antibody for S-glutathionylation was used. “?” nearby BH_4_ means that so far rather indirect evidence exists for eNOS uncoupling *via* BH_4_ depletion by GTN tolerance and noise exposure (*e.g.,* diminished GCH-1 and improvement of FMD by vitamin C or folic acid administration). Scheme in the *upper part* was reproduced from Knorr et al (2011) with permission. Images in the *lower part* were reproduced from Jabs et al (2015) and Knorr et al (2011) (for nitrate tolerance) and Munzel et al (2017a) (for noise exposure) with permission. Akt, protein kinase B; BH_4_, tetrahydrobiopterin; FAD, flavin adenine dinucleotide; FMD, flow-mediated dilation; FMN, flavin mononucleotide; GCH-1, GTP-cyclohydrolase-1; GSSG, glutathione disulfide; L-NAME, N^G^-nitro-l-arginine methyl ester; PKC, protein kinase C.

In addition to the classical activation mechanisms of eNOS by calcium/calmodulin, caveolin, heat shock protein 90, palmitoylation and myristoylation (Forstermann and Sessa, 2012), other processes such as phosphorylation and S-glutathionylation are involved in redox-active species formation (Forstermann and Munzel, 2006). “Redox switches” of eNOS are causing alterations of enzymatic eNOS activity and may contribute to the uncoupling of eNOS (Daiber et al, 2017a; Schulz et al, 2014), as depicted in Figure 2, in response to chronic GTN treatment or exposure to transportation noise. The precise definition of eNOS uncoupling is the process by which electrons leak from the transport within the reductase domain (from NADPH over flavin mononucleotide and flavin adenine dinucleotide) and are getting transferred to molecular oxygen leading to the formation of ^•^O_2_^−^ instead of ^•^NO.

This process is even more harmful than the consequences of inhibition of eNOS since uncoupling causes the enzyme to change from an ^•^NO to ^•^O_2_^−^ donor, thereby changing the biological environment from a vasodilator/antiatherosclerotic and thus beneficial to a harmful/proatherosclerotic phenotype (Forstermann and Munzel, 2006; Munzel et al, 2005b). Importantly, the uncoupling process is not only restricted to eNOS but may also involve neuronal NOS (type 1) (Vasquez-Vivar et al, 1999) as well as the inducible NOS (type 2) (Xia and Zweier, 1997), also reviewed in Daiber et al (2014).

The concept of eNOS uncoupling in GTN-induced nitrate tolerance was based on the “clinical” observation that in endothelium-denuded tolerant vessels, the potency of GTN was largely improved and ^•^O_2_^−^ formation in these vessels was significantly reduced (Munzel et al, 1995b), identifying the endothelium as a powerful ^•^O_2_^−^ source. We also established that the use of eNOS inhibitors such as N^G^-nitro-l-arginine decreased ^•^O_2_^−^ formation in nitrate-tolerant vessels (Knorr et al, 2011; Munzel et al, 2000b). Interestingly, similar observations were made in vessels from noise-exposed mice, where endothelial ^•^O_2_^−^ formation was significantly increased and normalized by pharmacological inhibition of eNOS by N^G^-nitro-l-arginine methyl ester (L-NAME) (Munzel et al, 2017a; Steven et al, 2020). The eNOS-derived ^•^O_2_^−^ formation was also accompanied by diminished vascular ^•^NO bioavailability, as depicted in Figure 2.

### Oxidative depletion of tetrahydrobiopterin

Among the concepts of eNOS uncoupling, the oxidative depletion of tetrahydrobiopterin (BH_4_) is the most commonly accepted one. In the 1990s, many preclinical as well as clinical studies demonstrated that the presence of BH_4_ is essential for a normal functioning of eNOS (Bendall et al, 2014; Kinoshita et al, 1997; Tsutsui et al, 1996). For example, Milstein and Katusic (1999) showed an oxidative degradation of BH_4_ to dihydrobiopterin (BH_2_) by peroxynitrite, thereby providing a concept of how RONS (especially peroxynitrite) *via* functional BH_4_ depletion may cause oxidative uncoupling of eNOS (Milstien and Katusic, 1999).

The oxidative BH_4_ to BH_2_ depletion concept also helped to explain why vitamin C is able to improve endothelial dysfunction in human subjects with various reasons for endothelial dysfunction as measured by plethysmography or flow-mediated dilation in chronic smokers (Heitzer et al, 1996), diabetic patients (Heitzer et al, 2001a), nitrate-tolerant subjects (Gori et al, 2010), and noise-exposed individuals (Herzog et al, 2019; Schmidt et al, 2013). Possible explanations may be the direct scavenging of ^•^O_2_^−^ or recycling of oxidized BH_4_ intermediates by vitamin C.

Although the reaction speed between vitamin C and ^•^O_2_^−^ (1.7 × 10^4^ *M*^−1^ s^−1^ for ascorbate) (Gotoh and Niki, 1992) is roughly 500,000-fold slower than the reaction speed between ^•^NO and ^•^O_2_^−^ (6.7–10 × 10^9^ *M*^−1^ s^−1^) (Kissner et al, 1997), the biological concentration of vitamin C is in the lower millimolar range in most intracellular biological fluids, the cytosol of most cell types (and at least 50–100 μ*M* in human plasma), and accordingly may compete with ^•^NO being present in the pico- to nanomolar range in most biological fluids (intra- and extracellular). The new concept postulates that the ^•^BH_4_^+^ radicals (once BH_2_ is formed, only energy-consuming enzymatic reaction confers reduction to BH_4_) (d'Uscio et al, 2003; Kuzkaya et al, 2003) will react with vitamin C to form BH_4_, which will replenish depleted BH_4_ stores thereby recoupling eNOS to an ^•^NO producing enzyme.

The essential role of BH_4_ for proper eNOS function was also proven by normalization of endothelial function in chronic smokers by supplementation with authentic BH_4_ (Heitzer et al, 2000). To exclude that solely the antioxidant properties of BH_4_ improved endothelial function, the smokers were also treated with tetrahydroneopterin (NH_4_), a structural analog of BH_4_ sharing the same antioxidant properties, which, however, failed to improve endothelial dysfunction in the smokers.

Likewise, supplementation with folic acid, which feeds into the *de novo* synthesis of BH_4_, improved endothelial function in nitrate-tolerant human subjects (Gori et al, 2001a). In addition, BH_4_ levels in vessels from tolerant rabbits were significantly decreased (Ikejima et al, 2008), whereas cotherapy of GTN-treated rats with the AT_1_-receptor blocker telmisartan normalized the aggravated eNOS-derived ROS formation and the diminished GTP-cyclohydrolase-1 expression (Knorr et al, 2011). The extent of the beneficial effect of vitamin C on endothelial function even turned out to be a valuable prognostic marker for cardiovascular events (predicting the risk for cardiovascular disease) (Heitzer et al, 2001b).

### S-glutathionylation of the eNOS reductase domain

**FIG. 3.**
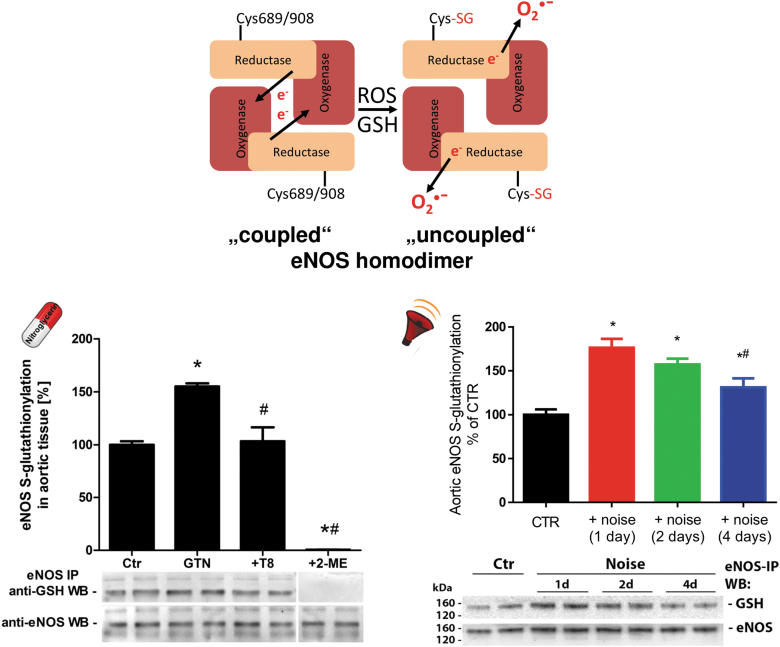
**S-glutathionylation of eNOS in GTN-treated nitrate-tolerant rats and aircraft noise-exposed mice.** *Upper part:* Oxidative stress causes S-glutathionylation of eNOS at cysteine 689 and 908, a surrogate marker for uncoupling of the protein. In the “coupled” eNOS homodimer, electrons are usually transferred from the NADPH and flavins to the heme iron. When cysteine residues 689 and/or 908 undergo S-glutathionylation, structural changes in eNOS are initiated that are followed by misdirection of the electrons to molecular oxygen with subsequent ^•^O_2_^−^ formation, termed “uncoupled” state of eNOS. *Lower part:* S-glutathionylation of eNOS was determined by eNOS immunoprecipitation from aortic tissue, followed by antiglutathione staining and normalization on eNOS. S-glutathionylation of eNOS was increased in nitrate-tolerant rats (GTN with a dose of 50 mg/kg/day for 3.5 days), which was normalized by telmisartan cotherapy (T8, 8 mg/kg/day for 10 days) (Knorr et al, 2011). *p < 0.05 versus Ctr, ^#^*p* < 0.05 versus GTN group. S-glutathionylation of eNOS was also increased by aircraft noise exposure [72 dB(A) around-the-clock for 1, 2, or 4 days] (Munzel et al, 2017a). Disappearance of the antiglutathione staining in the presence of 2-mercaptoethanol served as a control. *p < 0.05 versus Ctr, ^#^*p* < 0.05 versus noise (1 day) group. Representative blots are shown at the *bottom* of each densitometric quantification along with the respective loading control. Scheme in the *upper part* was adapted from Daiber et al (2020b) with permission. Data in the *lower part* were reproduced from Knorr et al (2011) (for nitrate tolerance) and Munzel et al (2017a) (for noise exposure) with permission.

S-glutathionylation is a rather novel and important redox regulatory mechanism for many enzymes, and S-glutathionylation of eNOS at one or more cysteine residues of the reductase domain was able to cause uncoupling of the enzyme (Fig. 3) (Chen et al, 2010). Cysteines 689 and 908 in the reductase domain have been identified as targets for redox modifications. Subsequently, Chen et al (2011) have demonstrated superoxide-induced thiyl radical formation in eNOS and the following intracellular disulfide formation or S-glutathionylation are both leading to uncoupling of eNOS. Meanwhile, eNOS S-glutathionylation was reported for a broad spectrum of pathophysiological conditions, including hypertension, diabetes, and atherosclerosis (for review, see Daiber et al, 2019b).

As we have shown, eNOS S-glutathionylation is largely increased in GTN-treated endothelial cells and aortic tissue from GTN-infused rats, likely contributing to eNOS uncoupling and endothelial dysfunction in the setting of nitrate tolerance, which was largely prevented by AT_1_-receptor blocker therapy with telmisartan (Fig. 3) (Knorr et al, 2011). This mechanism of eNOS uncoupling seems to be shared by other organic nitrates since isosorbide-5-mononitrate treatment of mice leads to enhanced aortic eNOS S-glutathionylation and endothelial ROS formation as well as endothelial dysfunction (Oelze et al, 2013). Likewise, eNOS S-glutathionylation, enhanced endothelial ROS formation, and endothelial dysfunction are the result of exposure of mice to aircraft noise around-the-clock (Fig. 3) (Munzel et al, 2017a), in particular, of exposure during the sleep phase (Kroller-Schon et al, 2018).

In addition, eNOS S-glutathionylation, ROS formation, and endothelial dysfunction were also exacerbated in an additive manner by noise exposure of hypertensive mice (Steven et al, 2020) or combined exposure to noise and particulate matter (Kuntic et al, 2023). Thus, we postulate that eNOS S-glutathionylation represents a “master redox switch” determining whether the enzyme produces ^•^NO or ^•^O_2_^−^. This pathomechanism is also of high interest from a redox perspective since eNOS S-glutathionylation is reversed by glutaredoxin-1 (Chen et al, 2013) and is tightly connected to BH_4_ deficiency (Crabtree et al, 2013).

### Additional mechanisms of eNOS uncoupling

Another direct redox-regulatory pathway explaining, at least in part, eNOS dysfunction due to eNOS uncoupling is the oxidative disruption of the zinc–sulfur complex (ZnCys_4_) in the binding region of the eNOS dimer. This will lead to a loss of eNOS dimerization, a phenomenon that has been first described by Zou et al (2002) with respect for ONOO^−^-mediated oxidation of eNOS. The critical role of this zinc–sulfur complex for proper eNOS dimer formation was demonstrated by a significant reduction of dimerization and thus more monomerization in a knockin mouse expressing a C101A-eNOS mutant with reduced zinc–sulfur complex building capacity (Suvorava et al, 2015). The reports on this “redox switch” reflected by a decreased eNOS dimer/monomer ratio were previously summarized and described for various disease conditions such as diabetes, hypertension, and hyperhomocysteinemia (Daiber et al, 2019b; Daiber et al, 2014; Schulz et al, 2014).

A diminished eNOS dimer/monomer ratio was also reported for GTN-treated endothelial cells, which could be prevented by pharmacological arginase-2 inhibition. In addition, nitrate tolerance was absent in aorta from animals chronically treated with GTN in the setting of genetic arginase-2 deficiency (Khong et al, 2012). However, this attractive uncoupling mechanism of eNOS was so far not detected in response to transportation noise exposure.

Another redox-sensitive regulatory pathway of eNOS activity represents the phosphorylation of the enzyme (Fig. 2). In general, there are three different important phosphorylation sites regulating the activity of eNOS. First, the activating phosphorylation at Ser1177 mediated by the protein kinase B pathway, which is calcium independent and increases the ^•^NO producing activity of eNOS (Dimmeler et al, 1999). Second, the inactivating phosphorylation at Tyr657 mediated by the protein tyrosine kinase-2 (PYK-2), which inhibits the enzyme without evidence for uncoupling of the enzyme (Loot et al, 2009). Third, the phosphorylation at Thr495 mediated by PKC, which may contribute to uncoupling and superoxide production by eNOS (Fleming et al, 2001; Lin et al, 2003).

It should be mentioned that the phosphorylation of eNOS at Thr495 or Tyr657 is initiated by increased oxidative stress. For example, the PKC-driven phosphorylation at Thr495 is stimulated by H_2_O_2_ (Lin and Takemoto, 2005; Rathore et al, 2008). The PYK-2-triggered phosphorylation at Tyr657 is stimulated by authentic H_2_O_2_ as well as by angiotensin-II induced ROS formation *in vitro* and *in vivo* (Loot et al, 2009). Thus, both the regulatory pathways may be regarded as initiators of “redox switches” of eNOS. Interestingly, PKC, which is activated in endothelial cells in response to GTN treatment, contributes to eNOS uncoupling *via* phosphorylation of eNOS at Thr495, a process that has been shown for being prevented by cotherapy with the AT1-receptor blocker telmisartan (Knorr et al, 2011).

Asymmetric dimethylarginine (ADMA) is probably the most potent endogenous inhibitor of eNOS (Boger, 2003a) and it is still controversial whether ADMA can cause uncoupling of the enzyme (Sydow and Munzel, 2003). Elevated ADMA serum/plasma levels have been established to represent a reliable risk marker (and probably promoter) concerning future cardiovascular events and prognosis in patients with established cardiovascular disease (Boger, 2003b; Schnabel et al, 2005). Oxidative stress within the vasculature significantly contributes to ADMA production and/or inhibition of ADMA degradation (due to redox sensitivity of protein arginine methyltransferases and dimethylarginine dimethylaminohydrolases [DDAHs]) (Daiber et al, 2014), leading to ADMA concentrations that will significantly inhibit eNOS activity (Cooke, 2000). For example, GTN increased ADMA levels in cultured human umbilical vein endothelial cells being associated with higher ROS production and malondialdehyde levels as well as diminished cGMP formation and mitochondrial ALDH-2 activity (Zhang et al, 2008).

However, higher ADMA levels in response to chronic GTN therapy could not be confirmed in a human study (Thomas et al, 2009). Whether chronic transportation noise will increase ADMA levels remains to be established.

l-Arginine is the endogenous substrate of eNOS for the ^•^NO synthesis. So it is not surprising that “l-arginine deficiency” has been proposed to contribute to uncoupling of eNOS (Bode-Boger et al, 2007). However, since the K_m_ of the enzyme (the concentration of l-arginine that is necessary for half maximal saturation) is ∼2.9 μ*M* for eNOS (Forstermann et al, 1991) and the intracellular l-arginine concentration is normally in the mM range, the concept that l-arginine depletion as a substrate is a significant regulator of eNOS activity is highly unlikely.

It remains also controversial whether l-arginine supplementation increases or decreases ROS formation from uncoupled NOS enzymes (reviewed in Daiber et al, 2014). Possible explanations for the beneficial effects of high concentrations of l-arginine may be either the direct antioxidant effects of the guanidino group in this amino acid (Lass et al, 2002) or an outcompeting of ADMA by l-arginine with respect to eNOS. The latter hypothesis becomes even more attractive if one considers the export of ADMA from endothelial cells that is driven by the cationic amino acid transporter-1 (CAT-1) in the presence of an extracellular excess of cationic amino acids such as l-arginine (Closs et al, 2012).

This concept has been described in a patient with frequent coronary artery spasms caused by a genetic defect in y^+^LAT expression, a major transporter for cationic amino acids, that may cause increased ADMA levels in the cytosol of endothelial cells and eNOS inhibition or uncoupling, all of which was corrected by administration of high-dose l-arginine, probably by CAT-1/l-arginine-driven export of ADMA from endothelial cells and recoupling of eNOS (Closs et al, 2012). As discussed above, the protective effects of pharmacological or genetic arginase inhibition in GTN-treated cultured cells or animals support a beneficial role of l-arginine in the setting of nitrate tolerance (Khong et al, 2012), whereas improvement of noise exposure damage by l-arginine still lacks experimental proof.

Also MPO was reported to control eNOS activity and/or nitric oxide bioavailability by direct reaction of the MPO compound 1 (highly oxidized iron intermediate during the reaction cycle of peroxidases) with nitric oxide, thereby consuming the vasodilator (Baldus et al, 2004; Eiserich et al, 2002; Rudolph et al, 2012) and thereby inducing endothelial dysfunction (Abdo et al, 2017; Stocker et al, 2004). In addition, there is a kind of pathophysiological cross talk between the eNOS inhibitor ADMA and MPO, in which ADMA activates neutrophils and the release of MPO that in turn suppresses DDAH (enzyme for breakdown of ADMA) and leads to endothelial dysfunction (von Leitner et al, 2011). Chlorinated reaction products of l-arginine and MPO were reported to inhibit eNOS activity (Zhang et al, 2001), and hypochlorous acid by vascular peroxidase 1 was also demonstrated to inhibit/uncouple eNOS enzyme (Liu et al, 2017).

## Identification of Uncoupled eNOS in Vascular Cells and Tissue

### Direct detection of eNOS-derived superoxide formation

According to previous reports on nitrate tolerance (Munzel et al, 2000a; Munzel et al, 2000b) and chronic transportation noise exposure (Kroller-Schon et al, 2018; Munzel et al, 2017a), ^•^O_2_^−^ formation from uncoupled eNOS is best identified by the measurement of ^•^O_2_^−^ formation in the presence and absence of NOS inhibitors. While inhibition of eNOS by eNOS inhibitors will consistently increase the ^•^O_2_^−^ signal in control tissues (excluding direct antioxidant effects of the NOS inhibitors), the use of eNOS inhibitors will decrease the ^•^O_2_^−^ signal in case eNOS is uncoupled and represents a significant ^•^O_2_^−^ source.

Methodologically, these studies used lucigenin in aortic tissue, a chemiluminescence dye that reacts with ^•^O_2_^−^ and forms a dioxetane product that decomposes to acridone and emits chemiluminescent light (Liochev and Fridovich, 1997). Lucigenin was suspected to undergo redox cycling and cause artificial ^•^O_2_^−^ production (Janiszewski et al, 2002; Tarpey et al, 1999). However, subsequent studies have shown that redox cycling of lucigenin requires artificial biological conditions (*e.g.,* exogenous addition of NADH) and that low lucigenin concentrations (5 μ*M*) are suitable for biological detection of ^•^O_2_^−^ (Daiber et al, 2004a). In particular, the close correlation between ^•^O_2_^−^ measurements by lucigenin and electron paramagnetic resonance (EPR) data wiped out the suspicion of artifact research by lucigenin.

Other suitable detection methods for NOS uncoupling (always in combination with a specific NOS inhibitor such as L-NAME) that can be used include the cytochrome c assay (SOD inhibitable signal) (Cai et al, 2005; Landmesser et al, 2003), high-performance liquid chromatography-based 2-hydroxyethidium quantification (the superoxide-specific oxidation product of dihydroethidium [DHE]) (Dikalova et al, 2010b; Xia et al, 2010), and DHE-dependent oxidative fluorescence microtopography in DHE-stained aortic cryosections [examples for nitrate tolerance (Jabs et al, 2015; Knorr et al, 2011) and noise exposure (Kroller-Schon et al, 2018; ; Munzel et al, 2017a; Steven et al, 2020)]. Representative staining images for the detection of eNOS uncoupling for nitrate tolerance by DHE-dependent oxidative fluorescence microtopography are shown in Figure 2, and the methodological details were explained in full depth previously (Daiber et al, 2014).

The different methods to detect vascular ^•^O_2_^−^ production have been reviewed in the past (Dikalov et al, 2007; Munzel et al, 2002) and were also discussed, highly controversial, either in favor (Daiber et al, 2017b) or in disfavor of these classical ROS detection assays (Griendling et al, 2016). As mentioned, EPR-based measurement of superoxide- or hydroxyl-adducts with different spin probes (*e.g.,* 5,5-dimethyl-1-pyrroline N-oxide-OOH or –OH adducts) demonstrated also high specificity for ^•^O_2_^−^, for example, in response to GTN treatment, although the sensitivity of these spin probes in vascular samples or cell culture is rather limited (Dikalov et al, 1998).

### Indirect detection of eNOS uncoupling

Indirect approaches to detect eNOS uncoupling are mainly based on testing the activity of eNOS (namely, NO formation or endothelium-dependent vasodilation) in the presence of drugs that are known to ameliorate the function (coupling state) of eNOS (*e.g.,* folic acid, sepiapterin, BH_4_) (Daiber et al, 2019b; Daiber et al, 2014). The substantial improvement of impaired endothelial function in chronic smokers by BH_4_ infusion is an important example for the recoupling of eNOS in a disease setting (Heitzer et al, 2000). In these investigations, one has to consider that BH_4_ is a strong antioxidant, making it difficult to differentiate whether the improvement of endothelial dysfunction is due to eNOS recoupling of just the consequence of the antioxidant properties of BH_4_.

Thus, as an internal control, the analog NH_4_ with comparable antioxidant properties such as BH_4_ has to be tested. For example, in the case of chronic smokers, BH_4_ improved endothelial dysfunction, but NH_4_ did not rule out unspecific antioxidant properties of the electron donor BH_4_.

Another example for indirect proof of an uncoupled eNOS was reported in the animal model of deoxycorticosterone acetate salt hypertension (Landmesser et al, 2003), showing the impaired ^•^NO synthesis by a decreased EPR-NO-signal and the increase of eNOS activity in response to supplementation with BH_4_ or by genetic deletion of the NADPH oxidase subunit *p47^phox^* (removing the “kindling radical” for NOS uncoupling). The prevention of endothelial dysfunction in isolated vessels of diseased animals (*ex vivo*) by the BH_4_ precursor sepiapterin is another approach to prove eNOS uncoupling in vascular tissue (Laursen et al, 2001; Schuhmacher et al, 2010).

**FIG. 4.**
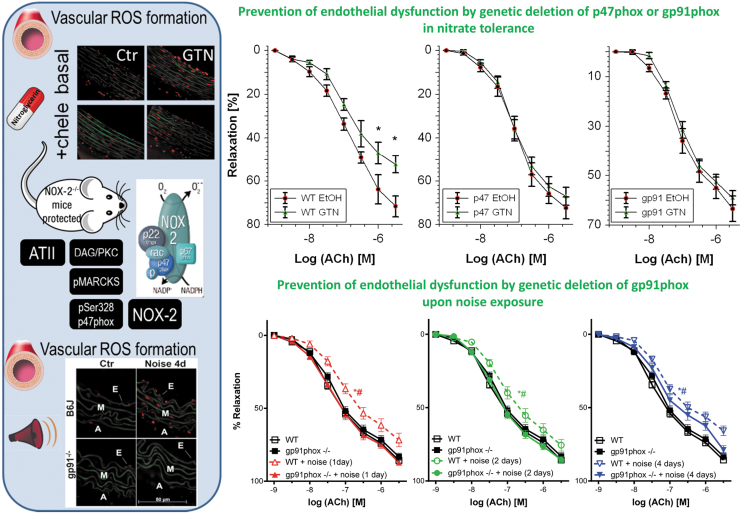
**Prevention of vascular ROS formation and endothelial dysfunction by GTN or traffic noise exposure by pharmacological or genetic NADPH oxidase inhibition.** *Left part:* Oxidative stress assessed by DHE staining (red fluorescence indicates reactive oxygen species [ROS] formation, whereas green fluorescence represents the autofluorescence of the basal lamina). E=Endothelium, M = Media, A = Adventitia. GTN (50 mg/kg/day for 3.5 days) treatment leads to aortic oxidative stress in rats, which was prevented by the PKC inhibitor chelerythrine (chele) (Knorr et al, 2011). Aircraft noise exposure [72 dB(A) around-the-clock for 1, 2, or 4 days] leads to aortic oxidative stress in rats, which was prevented by genetic deficiency of *gp91^phox^* (Kroller-Schon et al, 2018). The phagocytic NADPH oxidase (NOX-2) plays a key role in the pathomechanisms underlying nitrate tolerance and noise exposure-mediated cardiovascular damage. NOX-2 is activated by ATII receptor activation, leading to DAG formation, a strong PKC activator. PKC activation can be envisaged by phosphorylation of its target protein MARCKS. Activated PKC will cause phosphorylation of p47^phox^ at Ser328 leading to translocation of this cytosolic regulator of NOX-2 to the multienzyme membrane complex of gp91^phox^ with subsequent activation of NOX-2 and superoxide formation. *Right part:* Endothelial dysfunction (impaired ACh-dependent relaxation) by GTN treatment was prevented in mice with genetic deficiency of *p47^phox^* or *gp91^phox^* (Wenzel et al, 2008). *p < 0.05 versus WT EtOH solvent control group. Endothelial dysfunction by noise exposure was prevented in mice with genetic deficiency of *gp91^phox^* (Kroller-Schon et al, 2018). *p < 0.05 versus WT control group and ^#^*p* < 0.05 versus *gp91phox^−/−^* + noise group. Scheme in the *left part* was adapted from Frenis et al (2021c) with permission [Copyright © 2021 the authors. Open access (CC BY)]. Data in the *left part* were reproduced from Knorr et al (2011) (for nitrate tolerance) and Kroller-Schon et al (2018) (for noise exposure) with permission. Data in the *right part* were reproduced from Wenzel et al (2008) (for nitrate tolerance) and Kroller-Schon et al (2018) (for noise exposure) with permission. ACh, acetylcholine; ATII, angiotensin-II; DAG, diacylglycerol; DHE, dihydroethidine; ROS, reactive oxygen species.

We here provide indirect proof for eNOS uncoupling in the animal models of GTN-induced nitrate tolerance (Knorr et al, 2011; Wenzel et al, 2008) and aircraft noise exposure-mediated cardiovascular damage (Eckrich et al, 2021; Kroller-Schon et al, 2018) by normalization of vascular ROS formation and improvement of endothelial dysfunction by genetic deletion of subunits of the phagocytic NADPH oxidase (NOX-2), namely *p47^phox^* or *gp91^phox^*, or pharmacological inhibition of the enzyme (Fig. 4). The phagocytic NADPH oxidase (NOX-2) represents a major trigger of oxidative eNOS uncoupling due to its abundance in inflammatory cells (Wenzel et al, 2017). Vascular ROS formation in nitrate-tolerant animals under GTN therapy could be corrected by the NADPH oxidase inhibitor apocynin (Fukatsu et al, 2007) and the PKC inhibitor chelerythrine or others (Knorr et al, 2011; Munzel et al, 1995a).

Likewise, vascular ROS formation in noise-exposed mice was completely corrected by genetic deletion of *gp91^phox^* or pharmacological NADPH oxidase inhibitors such as GSK2795039 (Kroller-Schon et al, 2018). Importantly, *p47^phox^* or *gp91^phox^* knockout mice were protected from GTN-induced cross tolerance (endothelial dysfunction), whereas the degree of nitrate tolerance (the impaired vasodilatory potency of GTN) was not improved in the knockout mice (Fig. 4) (Wenzel et al, 2008). Similar observations were made for noise exposure-induced endothelial dysfunction that was completely prevented in *gp91^phox^* knockout mice upon 1 or 2 days of noise exposure, whereas upon 4 days of noise exposure, only partial protection by gp91^phox^ deficiency was observed, most likely because other ROS sources such as mitochondria take over after prolonged noise exposure (Fig. 4) (Kroller-Schon et al, 2018). Also noise-induced microvascular dysfunction enhanced the leukocyte–endothelium interaction, and the pro-atherothrombotic phenotype of the plasma proteome was prevented in* gp91^phox^* knockout mice (Eckrich et al, 2021).

However, as mentioned above, also other NOX isoforms may contribute to eNOS uncoupling in nitrate tolerance and transportation noise stress as detailed above for arterial hypertension in the angiotensin-II model. As for nitrate tolerance, downregulation of NOX-1 expression despite elevation of NADPH oxidase activity was reported for GTN-treated cultured smooth muscle cells (Szocs et al, 2007). *In vivo* GTN treatment of rats did not increase the expression of NOX-1 or NOX-4, neither at the protein nor gene level, and also, no increased ROS formation was observed by acute GTN treatment of cultured human embryonic kidney (HEK293) cells that were transfected with *Nox-1*, *Nox-4*, and the related other required subunits for full activation (Wenzel et al, 2008). *In vivo* treatment of mice with isosorbide-5-mononitrate increased NOX-1, NOX-2, and NOX-4 protein expression in the aorta, and NOX-4 protein expression was also increased in isosorbide-5-mononitrate-treated cultured primary human endothelial cells (Oelze et al, 2013).

As for noise-induced stress, aircraft noise exposure of mice increased aortic NOX-2 but not NOX-1 protein expression, whereas *Nox-1* mRNA levels were significantly increased in mouse lung endothelial cells (Munzel et al, 2017a). NOX-1 and NOX-4 protein levels were also increased by trend in the cortex of noise-exposed mice, whereas NOX-2 was almost not changed and NOX-3 expression was even decreased (Kroller-Schon et al, 2018). *Nox-1* mRNA was also upregulated in brains of mice upon sleep-phase noise exposure (Kroller-Schon et al, 2018). In addition, NOX-1 upregulation and NOX-3 downregulation were reported for the cochlea of rats exposed to loud noise in the context of hearing loss (Vlajkovic et al, 2013).

Transgenic mice overexpressing *Nox-4* showed more pronounced vulnerability to noise-induced hearing loss (Morioka et al, 2018), which was in accordance with the increase in NOX-4 expression in cochlea of noise-exposed mice (Shih et al, 2021). Genetic *Nox-3* deficiency protects from noise-induced sensorineural hearing loss (Rousset et al, 2022).

## Clinical Perspective on the Role of Endothelial Dysfunction as a Consequence of eNOS Uncoupling in Nitrate Tolerance and Transportation Noise Exposure

Multiple mechanisms may account for endothelial dysfunction in response to chronic nitrate therapy (Munzel et al, 2013; Munzel et al, 2011) and traffic noise exposure (Munzel et al, 2021; Munzel et al, 2018a), comprising modulation of the activity and/or expression of eNOS, reduced sensitivity of vascular smooth muscle cells to ^•^NO, or more pronounced scavenging of ^•^NO *via* its reaction with ^•^O_2_^−^. In case of inappropriately high ROS production and diminished vascular ^•^NO bioavailability and endothelial dysfunction, vascular responses are in general improved by high doses of the antioxidant vitamin C (Duffy et al, 2001; Heitzer et al, 1996) (also reviewed in Daiber et al, 2017c; Schulz et al, 2008), which can be attributed to a direct free radical scavenging effect, and also to the recycling of oxidized BH_4_-radicals by vitamin C (d'Uscio et al, 2003; Kuzkaya et al, 2003).

Importantly, the degree of endothelial function amelioration by vitamin C has prognostic implications (Heitzer et al, 2001b). There are numerous reports demonstrating the prognostic importance of endothelial function, for example, for patients undergoing peripheral or coronary bypass surgery (Gokce et al, 2002), as well as for patients with essential hypertension (Perticone et al, 2001).

**FIG. 5.**
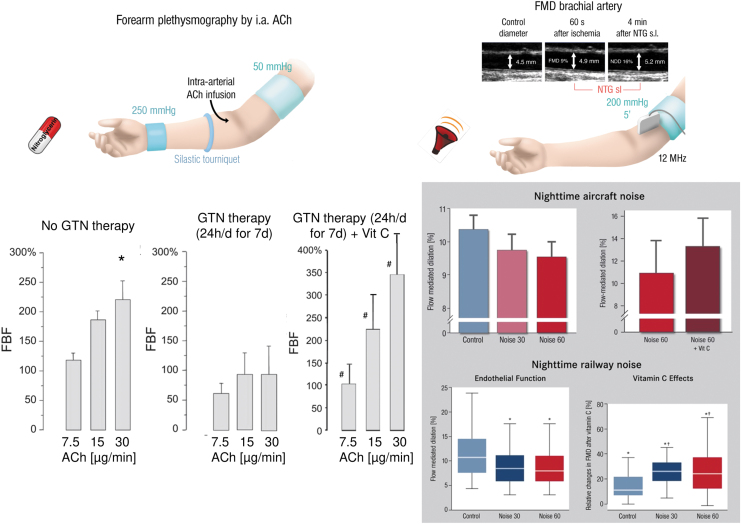
**Impairment of endothelial dysfunction by GTN therapy or traffic noise exposure and improvement by vitamin C coadministration.** *Left part:* Endothelial function was determined by forearm plethysmography, ultrasound-dependent assessment of forearm blood flow in response to increasing doses of infused ACh. FBF was expressed as the ratio of infused to noninfused arm. Each *column* presents the percent change in FBF from baseline in response to each infused concentration of ACh (7.5, 15, and 30 μg/min) in the untreated control group, chronic GTN treatment for 7 days, and the chronic GTN+vitamin C administration group (Gori et al, 2010). *p < 0.05 versus lowest ACh dose (7.5 μg/min), ^#^*p* < 0.05 versus GTN therapy without vitamin C. *Right part:* Endothelial function was determined by FMD, ultrasound-dependent assessment of vasodilation (widening) of a large vessel in the arm upon reactive hyperemia after vascular occlusion for 5 min. FMD was determined in subjects without aircraft noise exposure [L_eq_ 35.4 dB(A)] and 30 or 60 aircraft noise events for 1 night [L_eq_ 43.1 or 46.3 dB(A)] (Schmidt et al, 2013). Likewise, FMD was determined in subjects without train noise exposure [L_eq_ 33 dB(A)] and 30 or 60 train noise events for 1 night [L_eq_ 52 or 54 dB(A)] (Herzog et al, 2019). The effect of vitamin C oral administration on FMD was measured in both the noise exposure studies. *p < 0.05 versus same group without vitamin C, ^†^*p* < 0.05 versus unexposed control group with vitamin C. The schemes in the upper parts were reused from Daiber et al (2017c) with permission. Data in the *left part* were adapted from Gori et al (2010) (for nitrate tolerance) with permission. Data in the *right part* were adapted from Herzog et al (2019) and Schmidt et al (2013) (for noise exposure) with permission. FBF, forearm blood flow.

Chronic organic nitrate therapy causes endothelial dysfunction (impaired acetylcholine-dependent increase in forearm blood flow), all of which was completely prevented by coadministration of vitamin C (Fig. 5) (Gori et al, 2010). Also, several human studies documented that impaired endothelial function in response to aircraft or train noise exposure could be reversed by acute oral vitamin C administration (Herzog et al, 2019; Schmidt et al, 2013). In most instances, it is likely that an uncoupled eNOS contributes to endothelial dysfunction since the subsequent studies in animals were able to identify the uncoupling process in full detail, for example, as shown for nitrate tolerance (Knorr et al, 2011; Munzel et al, 2000a) and noise exposure (Daiber et al, 2020a; Kroller-Schon et al, 2018; Munzel et al, 2017a).

In summary, endothelial dysfunction, atherosclerosis, and the status of full-blown cardiovascular disease are associated with a chronic activation of the local and/or circulating renin–angiotensin–aldosterone system (RAAS) and an uncoupled eNOS. This was demonstrated in the setting of nitrate tolerance in response to chronic isosorbide-5-mononitrate therapy and GTN therapy, and also in response to transportation noise exposure, when the animals were exposed in the sleeping phase. Accordingly, in the setting of nitrate tolerance (Kurz et al, 1999) and noise stress (Daiber et al, 2019a; Munzel et al, 2021), inhibitors of the RAAS were able to improve nitrate tolerance, to reduce vascular oxidative stress, to normalize blood pressure, and to improve endothelial dysfunction most likely due to a recoupling of the eNOS enzyme.

**FIG. 6.**
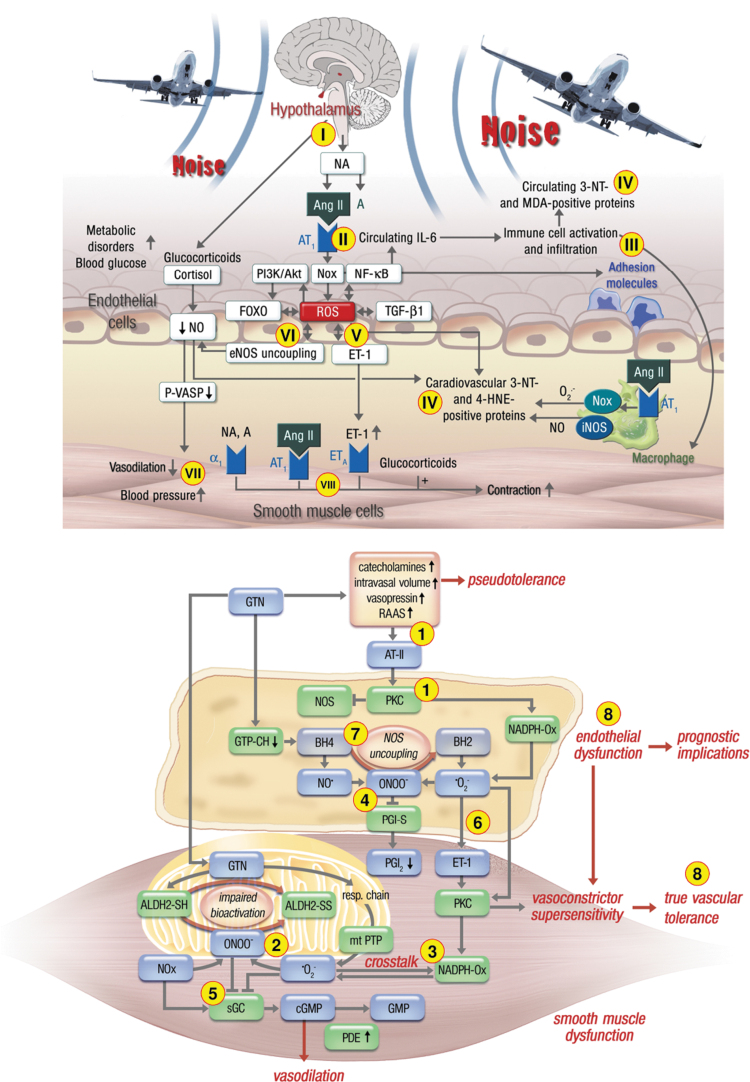
**Summarizing schemes on the similarities and differences of pathomechanisms underlying nitrate tolerance (*upper part*) and transportation noise exposure (*lower part*)-mediated cardiovascular damage.** (1) Chronic GTN therapy leads to activation of the RAAS *via* nitrovasodilator-induced hypotension with subsequent angiotensin-II release, PKC and NOX-2 activation (also called pseudotolerance). In addition, GTN therapy causes substantial mtROS formation also causing inhibition of ALDH-2, the GTN-bioactivating enzyme (2), followed by redox-dependent NOX-2 activation (3) and peroxynitrite (ONOO^−^) formation with subsequent dysregulation of other critical regulators of vascular tone by nitration of prostacyclin synthase (PGI-S) (4), oxidation of sGC (5), oxidative induction of ET-1 (6), and uncoupling of eNOS by BH_4_ depletion and S-glutathionylation (7), all of which leads to endothelial dysfunction and true vascular tolerance (8). In contrast, traffic noise exposure causes neuronal stress responses *via* the hypothalamic–pituitary–adrenal axis (release of cortisol/corticosterone) and the sympathetic nervous system (release of NA) (I) with subsequent RAAS and NOX-2 activation (II) along with enhanced inflammation (III). The resulting oxidative stress leads to peroxynitrite formation, protein tyrosine nitration, and lipid peroxidation (IV), as well as oxidative induction of ET-1 (V) and eNOS uncoupling (VI), all of which lead to endothelial dysfunction and hypertension (VII), in part, mediated by direct vasoconstrictor effects (VIII). The major differences of the pathomechanisms of nitrate tolerance and noise exposure are the pathways that lead to activation of the RAAS, the substantial adverse effects of GTN on mitochondrial function and mtROS formation, and the strong inflammatory component underlying noise exposure-mediated damage. The major similarities are the central role of oxidative stress in both pathomechanisms of endothelial dysfunction, with NOX-2, RAAS, and eNOS uncoupling as key players. The schemes were reused from Munzel et al (2011) (*upper part*) and Munzel et al (2017a) (*lower part*) with permission. ALDH-2, mitochondrial aldehyde dehydrogenase; ET-1, endothelin-1; mtROS, mitochondrial reactive oxygen species; NA, noradrenalin; RAAS, renin–angiotensin–aldosterone system; sGC, soluble guanylyl cyclase.

Nitrate-tolerant animals and patients take profit from AT_1_-receptor blockade (Hirai et al, 2003; Knorr et al, 2011) and angiotensin-converting enzyme (ACE) inhibitor (Berkenboom et al, 1999; Heitzer et al, 1998; Watanabe et al, 1998) therapy. Similar observations were made in noise exposure animal studies, where RAAS activation was prominent as documented by elevated angiotensin-II levels (Munzel et al, 2017a), additive damage of noise in mice with arterial hypertension by angiotensin-II infusion (Steven et al, 2020), and normalization of elevated blood pressure in noise-exposed mice by the ACE inhibitor captopril (Frenis et al, 2021b). Although the molecular mechanisms of RAAS activation and increased ROS production are likely different in response to chronic nitrate therapy and noise exposure, the downstream health side effects on the cardiovascular system are likely the same (see summarizing scheme in Fig. 6).

Organic nitrate therapy induces neurohormonal (counter-regulatory) activation of vasoconstrictor pathways including the RAAS as a consequence of GTN-induced hypotension, but this response may be also redox-driven as a consequence of increased endothelin-1 or angiotensin-II expression/release and signaling (Munzel et al, 2013; Munzel et al, 2011). In contrast, transportation noise induces RAAS activation *via* upstream induction of stress response pathways such as the hypothalamic–pituitary–adrenal axis, leading to increased cortisol release and activation of the sympathetic nervous system that are known to be tightly connected to downstream vasoconstrictor pathways based on endothelin-1 or angiotensin-II (Daiber et al, 2020a; Daiber et al, 2019a). Especially, the release of catecholamines such as noradrenaline by sympathetic activation may contribute directly to endothelial dysfunction by direct vasoconstriction *via* α_1_-receptor activation as well as downstream activation of RAAS and endothelin-1 pathway.

In the setting of nitrate tolerance, the hypotensive effects of organic nitrates and increase of intravasal volume can induce counter-regulatory effects, for example, elevated levels of noradrenaline reported for chronic GTN therapy (Parker et al, 1991) or acute nitrate infusion (Kubo et al, 1999). Likewise, chronic road noise exposure was associated with higher levels of noradrenaline in healthy women (Babisch et al, 2001), and acute exposure of healthy subjects to one night of aircraft noise caused elevated adrenaline levels (Schmidt et al, 2013), which was also supported by a chronic *versus* acute noise exposure study of humans (Ising and Braun, 2000) as well as animal studies (Munzel et al, 2017a; Said and El-Gohary, 2016). Catecholamines are interconnected with the RAAS and endothelin-1 pathway (reviewed in Daiber et al, 2020a; Daiber et al, 2019a; Munzel et al, 2021), with a key role for ROS derived from NADPH oxidases (Grande et al, 2011).

The similarities between the pathomechanisms underlying endothelial dysfunction in response to chronic noise exposure and GTN treatment-induced cardiovascular damage are evident (Fig. 6). They include the activation of a primary ^•^O_2_^−^ source such as the phagocytic NADPH oxidase (NOX-2) to produce the “kindling radicals” leading to uncoupling of eNOS *via* the above-described “redox switches” (Schulz et al, 2014). Upon RAAS activation, angiotensin-II receptor-mediated diacylglycerol formation and the subsequent PKC activation will lead to NOX-2 (phagocytic NADPH oxidase) activation. Likewise, there is an interaction between endothelin-1 and NOX-2 (Daiber et al, 2017a).

As a major difference of GTN therapy, NOX-2 activation could also be promoted by redox activation by mtROS generated by the interaction of the organic nitrate with the mitochondrial respiratory chain components (Daiber et al, 2004b; Munzel et al, 2013; Sydow et al, 2004). As a major difference of noise exposure, there is a strong inflammatory phenotype of tissues and the circulation, which was observed in noise-exposed human subjects (Herzog et al, 2019) and mice (Eckrich et al, 2021; Frenis et al, 2021a; Munzel et al, 2017a). In addition, noise exposure during the sleep phase induces sleep fragmentation and deprivation leading to dysregulation of the circadian clock (Daiber et al, 2022; Kroller-Schon et al, 2018; Munzel et al, 2020), which is *per se* an important cardiovascular risk factor (Lecour et al, 2022).

What mitigation strategies in the population could be applied against the cardiovascular complications by transportation noise exposure and chronic GTN and also isosorbide-5-mononitrate and dinitrate therapy-induced nitrate tolerance? First, as discussed above, RAAS inhibition (*e.g.,* by ACE inhibitors such as captopril or AT1-receptor blockers such as telmisartan) can be used to avoid the mentioned complications such as eNOS uncoupling, and several clinical studies confirmed that inhibitors of the RAAS can indeed develop nitrate tolerance (Mehra et al, 1992). This, however, does not represent a mitigation strategy that can be applied to the general population.

Second, acute vitamin C administration improved endothelial dysfunction induced by noise or GTN therapy, but we know from large-scale clinical trials that chronic oral vitamin C administration is mostly not protective because of the rather slow reaction rate between the antioxidant and superoxide (Gori and Munzel, 2011; Schmidt et al, 2015b), except few studies where vitamin C plasma levels were well controlled (Khaw et al, 2001).

We have shown in animal models of nitrate tolerance and aircraft noise exposure, activation of the endogenous antioxidant defense system *via* nuclear factor erythroid 2-related factor 2 (NRF2)/heme oxygenase-1 activation (*e.g.,* by hemin or dimethyl fumarate) (Bayo Jimenez et al, 2021; Schuhmacher et al, 2010; Wenzel et al, 2007), which represents a potential mitigation strategy that could be applied to large populations, for example, by dietary activators of NRF2 such as sulforaphane from broccoli and other nutraceuticals that are currently tested in clinical trials (Cuadrado et al, 2018). This approach could be promising since most environmental stressors induce damage that is responsive to NRF2 activators (Bayo Jimenez et al, 2022). Also, physical activity could be a first-line mitigation measure as cardiovascular damage by other environmental risk factors, for example, air pollution (Hahad et al, 2021; Tainio et al, 2021), is highly responsive to this nonpharmacological intervention.

Although there is evidence for improvement of noise adverse health effects by physical activity (*e.g.,* by proximity of green space areas), large clinical trials are not available. Wearing earplugs and noise-canceling headphones (especially at night) may in theory be effective, however, large clinical studies on the protective effects of earplugs and noise-canceling headphones against (nighttime) noise exposure are currently not available. Obviously, there are still many gaps in noise research that should urgently be addressed in light of the potential additive risk of noise exposure in individuals with preexisting cardiovascular disease suggested by human (Schmidt et al, 2015a) and animal studies (Steven et al, 2020), most probably by sleep deprivation/fragmentation-mediated circadian rhythm impairment by nighttime noise (Daiber et al, 2022).

## Abbreviations Used

8-oxoGua: 8-oxoguanine
ACE: angiotensin-converting enzyme
ACh: acetylcholine
ADMA: asymmetric dimethylarginine
Akt: protein kinase B
ALDH-2: mitochondrial aldehyde dehydrogenase
ATII: angiotensin-II
BH_2_: dihydrobiopterin
BH_4_: tetrahydrobiopterin
CAT-1: cationic amino acid transporter-1
CI: confidence interval
DAG: diacylglycerol
dB(A): decibel (A-weighted)
DDAH: dimethylarginine dimethylaminohydrolase
DHE: dihydroethidium
EDRF: endothelium-derived relaxing factor
eNOS: endothelial nitric oxide synthase (NOS-3)
EPR: electron paramagnetic resonance
ET-1: endothelin-1
FBF: forearm blood flow
FMD: flow-mediated dilation
FMN: flavin mononucleotide
GCH-1: GTP-cyclohydrolase-1
GPx: glutathione peroxidase
GR: glutathione reductase
GSSG: glutathione disulfide
GTN: nitroglycerin
iNOS: inducible nitric oxide synthase (NOS-2)
L-NAME: N^G^-nitro-l-arginine methyl ester
MPO: myeloperoxidase
mtROS: mitochondrial reactive oxygen species
NA: noradrenalin
NH_4_: tetrahydroneopterin
nNOS: neuronal nitric oxide synthase
NOX-1/-3/-4: NADPH oxidase isoforms 1/3/4
NOX-2: phagocytic NADPH oxidase (gp91phox)
NRF2: nuclear factor erythroid 2-related factor 2
P47^phox^: cytosolic/regulatory subunit of NOX-2
PKC: protein kinase C
PYK-2: protein tyrosine kinase-2
RAAS: renin–angiotensin–aldosterone system
RONS: reactive oxygen and nitrogen species
ROS: reactive oxygen species
sGC: soluble guanylyl cyclase
SOD: superoxide dismutase
TR: thioredoxin reductase
Trx: thioredoxin
ucNOS: uncoupled nitric oxide synthase
WHO: World Health Organization
XO: xanthine oxidase
